# Fungal Quorum-Sensing Molecules and Inhibitors with Potential Antifungal Activity: A Review

**DOI:** 10.3390/molecules24101950

**Published:** 2019-05-21

**Authors:** Arshad Mehmood, Guorong Liu, Xin Wang, Guannan Meng, Chengtao Wang, Ya Liu

**Affiliations:** 1 Beijing Advance Innovation Center for Food Nutrition and Human Health, Beijing Engineering and Technology Research Center of Food Additives, Beijing Technology and Business University (BTBU), Beijing 100048, China; arshadfst@yahoo.com (A.M.); linda19727651@163.com (X.W.); mgn123456mgn@163.com (G.M.); 2 R&D Center of China Tobacco Yunnan Industrial Co. Ltd., Kunming 650202, China; 13888060463@163.com

**Keywords:** quorum sensing, fungi, quorum-sensing molecules, quorum-sensing inhibitors, antifungal activity

## Abstract

The theory of persisting independent and isolated regarding microorganisms is no longer accepted. To survive and reproduce they have developed several communication platforms within the cells which facilitates them to adapt the surrounding environmental changes. This cell-to-cell communication is termed as quorum sensing; it relies upon the cell density and can stimulate several traits of microbes including biofilm formation, competence, and virulence factors secretion. Initially, this sophisticated mode of communication was discovered in bacteria; later, it was also confirmed in eukaryotes (fungi). As a consequence, many quorum-sensing molecules and inhibitors have been identified and characterized in various fungal species. In this review article, we will primarily focus on fungal quorum-sensing molecules and the production of inhibitors from fungal species with potential applications for combating fungal infections.

## 1. Fungal Quorum Sensing: An Over View

Microorganisms are no longer deemed as independent, isolated cells; apart from their survival and reproduction competition, they have evolved a communication process that enables them to adapt to local environmental changes [1,2,3]. The cell density of their population is an example of this, where to efficiently harness biological effects, communicating microorganisms coordinate their gene expression profiles under appropriate local cell densities [4]. Quorum sensing (QS) is a process in which links gene expression to the cell density of microbial populations. QS relies on the production and release of small diffusible chemical signaling molecules in the extracellular environment [5,6].

Pheromones or autoinducers are signaling molecules embroiled in cell–cell communication and considered as quorum-sensing molecules (QSM). These molecules act as transcriptional regulators; when the concentration hits a pivotal threshold (commensurate to a particular cell density), they bind to receptor molecules, which are located either on the surface or in the cytoplasm [1,6,7]. Transcriptional regulators then prompt or restrain the QS target gene and activate the genes encoding for quorum-sensing signal (QSS) synthesis, thereby generating a positive feedback loop, self-inducing an increase in the production of the corresponding signaling molecule [8]. Therefore, quorum sensing (QS) provides multicellular characteristics for bacteria and other microorganisms, i.e., characteristics associated with higher organisms [9].

**Figure 1:**
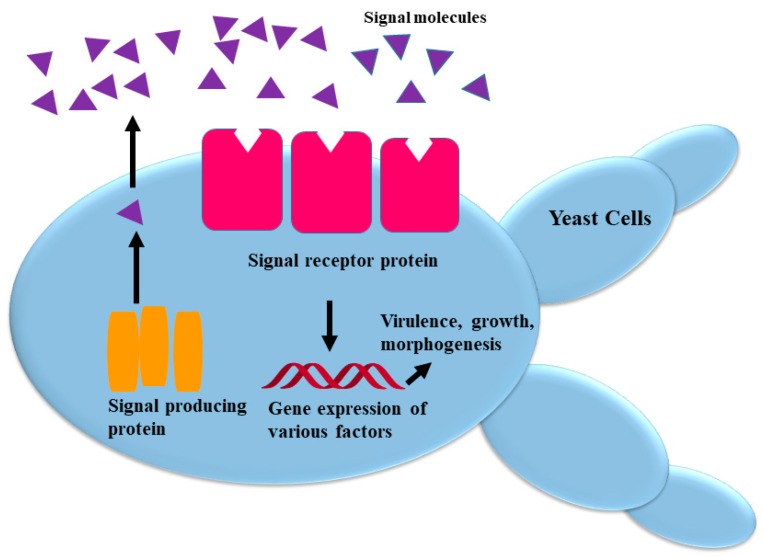
Quorum-sensing mechanism in Fungi (adapted from [3]). Signal molecules are synthesized from signal-producing proteins which are detected by signal-receptor proteins and stimulate various genes expression.

It was later discovered that quorum sensing is also ubiquitous in various fungal species. Single cells (yeast) and filamentous cells are two growth forms; fungal dimorphism or polymorphism is an environmental interconversion between yeast and mycelial morphology, which is manifested by some fungi (Figure 1) [1,2,3,6]. Albuquerque and Casadevall [10] detailed five criteria that must be fulfilled for a compound to be classified as a signaling molecule or QSM in bacteria or fungi. The molecule should (I) accrue in the extracellular environment throughout microbial growth; (II) amass in a concentration that is proportional to the density of the population cells, the influence of which is limited to a specific growth phase; (III) result in a coordinated response across the population, which is just an adaptation to metabolize or detoxify the molecule itself after reaching the threshold concentration; (IV) when appended to the culture exogenously, propagate the QS phenotype; and (V) is not merely a by-product of microbial catabolism. The characteristics of mainly QS fungal species, their mode of action, QSMs, QSIs (quorum sensing inhibitors), and their potential antifungal activities are briefly described below.

## 2. Quorum Sensing in Various Fungal Species

### 2.1. Candida Albicans

*Candida albicans* is a dimorphic yeast, and best known for many life-threating diseases. *C. albicans* are well studied for QS compared with other fungal species [2,11]. It undergoes various transformations, e.g., converting from a yeast to hyphal cell morphology under specific conditions. These changes are important for its adaptation to the environment and pathogenicity, and are governed by many environmental signals, e.g., carbon dioxide level, temperature, chelating agent, pH, cell density, nutrients composition and concentration [12].

Farnesol and tyrosol, two signaling compounds produced by *C. albicans* have been reported as QSMs with opposite effects. Farnesol, causes the switch from yeast to hyphae and yeast growth whereas tyrosol accelerates hyphal development and germ-tube formation. Farnesol-mediated morphological changes in *C. albicans* are complex but well studied [13,14,15]. Farnesol controls hyphal development via suppressing the Ras1-cAMP/protein kinase A (PKA) signaling pathway. The study reported that this compound also affects filamentation by repression of the cAMP pathway via inhibiting Cyr1 (adenylyl cyclase). It also down-regulated *Nrg1* and repressed hyphal development. Farnesol reverted Cup9 (a transcriptional repressor of negatively regulating the yeast to filamentous conversion) degradation by Ubr1 (N-end rule E3 ubiquitin ligase), Tup1 (a transcriptional repressor of hypha-specific genes), Chk1 (histidine kinase), and Czf1 (zinc finger 1), important response factors mediated by farnesol which further facilitate morphology changes [16,17,18,19,20,21].

### 2.2. Debaryomyces Hansenii

*D. hansenii* is a yeast that belongs to the *Saccharomycetaceae* family and is also reported to possess QS. It has the ability to grow in high salt concentrations with low temperature and pH which make them the best candidate for application in the food and cheese industry [22]. Earlier, Cruz et al. [23] reported that during continuous fermentation of acid-hydrolyzed barley bran, this yeast shows dimorphism. Later, another study also observed ammonia-mediated QS regulation in this species [24].

### 2.3. Cryptococcus Neoformans

**Figure 2:**
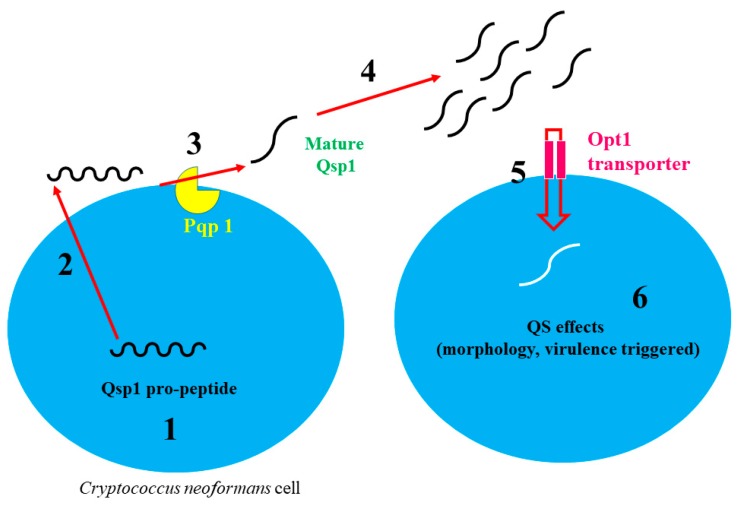
Quorum-sensing pathway in *Cryptococcus neoformans* (adapted from [26,31]). (1) Pro-peptide (QSP-1) is produced; (2) QSP-1 is excreted outside of the fungal cell; (3,4) it is broken down by protease Pqp1 and converted into mature (Qsp1 peptide); after that, (5) it is transported back into cells through Opt1 (oligopeptide transporter), and (6) it stimulates virulence and morphological changes.

*C. neoformans* is well known for meningoencephalitis and it is reported that, annually, around 625,000 deaths occur due to this species [25,26]. Previously, Lee et al. [27] reported a QS-like behavior in mutant *C. neoformans* serotype D strain deficient Tup1 co-repressor. They reported that mutant *tup1∆* strains did not grow when cells were plated at low numbers (<10^3^ cells/mL). However, with the addition of conditional medium (CM), growth was recovered. Moreover, they isolated 11 amino acid peptides from the supernatants cultured with *tup1∆* strain. They postulated that this peptide (*Qsp1*) may be responsible for a QS-like effect in *C. neoformans.* Later, the same research group described that the Tup1 QS effect was not common and not observed in serotype A, Tup1 mutant’s strains of *C. neoformans* [28]. Later, in another study, Madhani [29] described QS-like behavior in *C. neoformans,* and they observed that QS-like peptide (QSP1-4) was excreted outside the cell and then transferred into cell by the Opt1 transporter. In another study, a QS mechanism of phenotype Qsp1 mutant strains was described by Homer et al. [26]. They stated that Qsp1 peptide played a central role in the signaling and control of virulence in *C. neoformans*. Qsp1 mediates autoregulatory signaling that modulates secreted protease activity and promotes cell wall function at high cell densities. Peptide production requires release from a secreted precursor, proQsp1, by a cell-associated protease (Pqp1). Qsp1 sensing requires an oligopeptide transporter, Opt1, and remarkably, cytoplasmic expression of mature Qsp1 complements multiple phenotypes of *qsp1D*. Thus, *C. neoformans* produces an autoregulatory peptide that matures extracellularly but functions intracellularly to regulate virulence (Figure 2). Recently, Tian et al. [30] also reported that Qsp1 played an important role in the sexual and bisexual reproduction of *C. neoformans*. They reported that zinc finger regulator Cqs2 played an important role in the Qsp1 signaling cascade during unisexual and bisexual reproduction of *C. neoformans.*

### 2.4. Saccharomyces Cerevisiae

**Figure 3:**
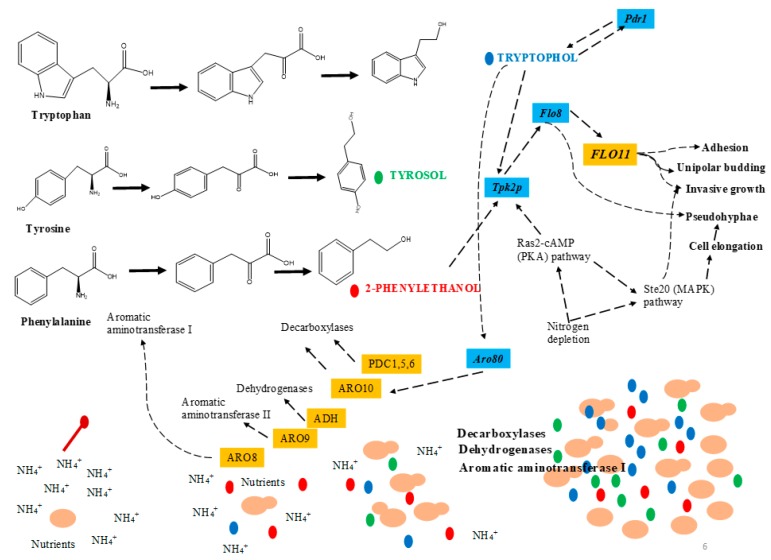
Quorum-sensing mechanism in *Saccharomyces cerevisiae* (adapted from [35]).

QS-like behavior of *S. cerevisiae* has been effectively reported by many authors [8,32,33,34,35]. In *S. cerevisiae,* tryptophol, tyrosol, and phenlyethanol are synthesized from the corresponding amino acids (tryptophan, tyrosine and phenylalanine) via the Ehrlich pathway (transamination, decarboxylation and reduction) under a low-nitrogen environment [36]. This pathway is reliant upon several factors, e.g., oxygen, pH, growth condition, and ammonia salt [37] (Figure 3).

Aromatic alcohols, tryptophol, and phenlyethanol collaboratively influence the *FLO11* up-regulation by cAMP-dependent PKA and *Flo8p* transcription factor [38]. The *FLO11* product (*Flo11p*) is vital for filamentous growth which adheres to the GPI (glycosylphosphatidylinositol) cell surface flocculin protein [39]. Chen and Fink [38] observed that deletion of *FLO8* or *TPK2* in the *S. cerevisiae* strain did not produce filaments in response to the aromatic alcohols. In another study, Wuster et al. [33] speculated that *MIG1* and *CAT8* were the key transcriptional regulator genes which were involved in the expression of the aromatic alcohol genes in *S. cerevisiae*

In the Ehrlich pathway, transamination occurs by enzyme aminotransferases I & II encoded by *ARO8* and *ARO9* genes whereas in the decarboxylation step, enzyme decarboxylase and pyruvate decarboxylases (encoded by *ARO10*) catalyze the reaction [40]. It was observed that the gene, *ARO8*, was the first to be activated in the early growth phase whereas *ARO9* and *ARO10* genes were activated at the start of the stationary phase. Furthermore, they also observed a close link between *ARO* genes expression and the production of QSMs [34]. Moreover, QSM production was also affected by several factors such as cell density, ethanol, nitrogen content, and aerobic/anaerobic growth conditions. QS also triggered phenotype alternations in *S. cerevisiae* that have a further influence on the rate of production of QSMs [35]. Although QS in *S. cerevisiae* is well studied, some important questions still need to be answered [35].

### 2.5. Neurospora Crassa

QS in *N. crassa* has, to date, not been well studied, and only Roca et al. [41] have shed light on it. They speculated that specialized hyphae (conidial anastomosis tubes) formation in *N. crassa* were reliant upon cell density. These are associated with hyphal fusion and varied between germ tubes. Moreover, they observed that the formation of conidial anastomosis tubes was depleted at low conidial concentrations; this trait was reliant upon mitogen-activated protein (MAP) kinase signaling/putative transmembrane protein but not on the *cAMP* signaling pathway.

### 2.6. Penicillium Species

The genus Penicillium is a popular fungal genus due to its ubiquity. It is observed in food spoilage, antibiotics, and mycotoxin production. The QS mechanism of the Penicillium *species* (P. sclerotiorum and P. decumbens) was reported by Raina et al. [42] and Guo et al. [43]. Raina et al. [42] documented that exogenous addition of multicolic acid (γ-butyrolactone-containing molecules) in P. sclerotiorum cultures stimulated the rate of production of sclerotiorin (a yellow, chlorine containing pigment possessing phospholipase A2 inhibitor activity). They discovered that multicolic acid (γ-butyrolactone-containing molecules) act as QSMs in P. sclerotiorum. Similarly, Guo et al. [43] observed that the addition of exogenous farnesol enhanced hyphal growth of P. decumbens, which resulted in higher secretion of cellulose.

### 2.7. Aspergillus Species

Previously, QS in *Aspergillus* species (*A. flavus, A. nidulans*, *and A. *terreus**) were studied by several authors [44,45,46,47]. Oxylipins in *A. flavus* [44,48] and *A. niger*, linoleic acid (*A. terreus*) [45], γ-heptalactone (*A. nidulans*) [49], and butyrolactone (*A. terreus*) [46] act as a QSM. QS in *Aspergillus* species remains a challenge for researchers due to the nature of these fungi. Further, mechanistic studies are needed to elucidate the putative QS mechanism of these fungi.

## 3. Quorum Sensing Molecules (QSMS)

### 3.1. Pheromones

**Figure 4:**
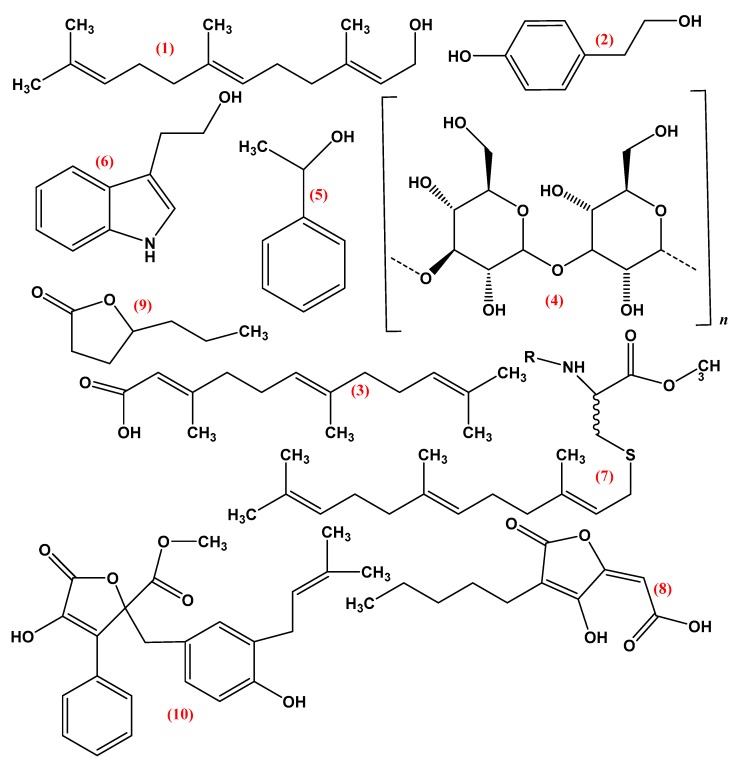
Chemical structure of various fungal quorum sensing molecules (1) Farnesol, (2) Tyrosol, (3) Farnesoic acid, (4) α-(1,3)-glucan, (5) 1-Phenyl-ethanol, (6) Tryptophol, (7) Pheromones 1. a-factor 2. α-factor, (8) Multicolanic acid, (9) γ-Heptalactone, (10) Butyrolactone-I.

Pheromones in fungi serve as QSMs. *S. cerevisiae* secreted pheromone peptides (a and α factor), these factors were produced by a and α cells. Individual mating types only produce one pheromone factor, which depends on MAT locus availability (Figure 4). These secreted pheromones accumulated and began to diffuse through the environment, where they are recognized by Ste2p and Ste3p G-protein receptors. Binding with pheromones results in the breakage of the α cascade by Ste5p and two phosphorylated MAP kinases, Fus3p and Kas1p. Furthermore, Fus3p enhanced the expression genes by the activation of the Ste12p transcription factor in the nucleus. Subsequently, as a phenotypic morphological response to the opposite mating pheromone, cells develop a shmoo, which is basically a directional growth of the cell in response to the pheromone gradient and results in plasmogamy between the opposite cells [50].

### 3.2. Farnesol

Farnesol is secreted by *C. albicans* during the sterol synthetic pathway via dephosphorylation of farnesol pyrophosphate (FPP). It is predicted that around 1.6% of all FPP are involved in farnesol production. There is a large variation regarding farnesol production in *C. albicans*. FPP is a forerunner for dolichol and ubiquinone and plays an important role in ergosterol biosynthesis pathway. The *DPP3* gene was found to be a key gene in *Candida* for this phosphatase production and knock out of this gene results in a decrease in the rate of production of farnesol [51]. Moreover, it was observed that in *S. cerevisiae*, farnesol was excreted into the medium when the *ERG9* gene was deleted, resulting in the accumulation of intracellular farnesol. Therefore, in these mutants, the production of farnesol was affected by the growth condition, resulting in a farnesol accumulation of 50 to 100 mg/L, where, as in case of *C. albicans*, it is directly secreted into the medium under standard culture conditions [52]. It was also observed that addition of Zaragozic acid B resulted in an eight-fold increase in farnesol production whereas azole antifungals, ketoconazole, clotrimazole, fluconazole, and miconazole resulted in a 10- to 45-fold increase in farnesol production. Therefore, inhibition or deletion of ERG9 (squalene synthase) results in accumulation of FPP and thus an increase in the farnesol concentration [53]. There are three reported pathways of farnesol production in *C. albicans*; (1) Chk1p MAP Kinase, (2) Ras-cAMP, and (3) Tup1 pathway [16,17,20]. Although several signaling pathways have been implicated in farnesol sensing, none of these has been shown to involve a specific farnesol receptor. It is possible that some of the identified signaling pathways might be related to nonspecific actions of farnesol. Due to the lipophilic nature of farnesol, it is possible that such a putative receptor is extracellular, membrane integrated, or cytosolic in nature. Until a specific farnesol receptor has been identified, it is unclear whether farnesol sensing is a specific receptor-mediated response [54].

### 3.3. Tyrosol

Tyrosol (2-[4-hydroxyphenyl] ethanol) is the derivative of tyrosine and the second reported QSM in *C. albicans*. Furthermore, it is also present in olive oil (Figure 4) [1,10,21,55,56]. It is discharged into the medium and shortens the lag time of cells to begin germination. It also accelerates the hyphal development [13]. Nickserson et al. [57] reported that tyrosol is a minor QSM and is only needed when farnesol is limited or absent. Similarly, Alem et al. [56] also confirmed its role as a QSM and its significant effect on biofilm formation.

### 3.4. Volatile Organic Compounds

Approximately 250 volatile organic compounds have been characterized in various fungal species. Previously, many researchers reported that these volatile compounds act as QSMs in fungi [58,59,60]. For example, previously, Palkova et al. [61] noticed the growth of *S. cerevisiae* on complex media form a turbid path with respect to neighboring colonies. They observed that this process was mediated by small volatile compounds which were required for the uptake of amino acid for its production. Interestingly, inactivation of SHR3 (a protein responsible for the correct localization of several yeast amino-acid permeases) breaks the turbid path between colonies. In another study, Nemcovic et al. [62] observed that volatile compounds (3-octanone, 3-octanol, and 1-octen-3-ol) induced conidia formation in *Trichoderma* spp. During conidiation formation, three 8-carbon compounds were produced. At a higher concentration (500 µM), all of these compounds suppressed the growth and conidiation formation in *Trichoderma* spp. However, only one compound (1-octen3-ol) remarkably suppressed its growth at a lower concentration (0.1 µM) [56,59].

### 3.5. Lactone Containing Molecules

The lactone-containing molecules butyrolactone-I, γ-butyrolactone, multicolanic, multicolosic, and multicolic acids have been reported to act as QSMs in fungal species, e.g., *A. terreus*, *A. nidulans*, and *P. sclerotiorum* (Figure 4) [46,47,62,63,64,65]. Butyrolactone-I is excreted as a secondary metabolite by *A. terreus*, and due to its small concentration, acts as self-regulating factors in some bacteria. It was observed that, by the addition of butyrolactone-I into the medium of *A. terreus*, hyphal branching increased three-fold, secondary metabolism occurred and submerged sporulation accelerated [63,64]. In another study, Schimmel et al. [63] reported that butyrolactone-I acted as a QSM in *A. terreus* because the addition of this molecule stimulated the production of secondary metabolites sulochrin (two-fold increase) and lovastatin (three-fold increase). These secondary metabolites have multiple beneficial functions in humans. They possess anti-cancer and anti-tumor functions and are inhibitors of cyclin-dependent kinase (CDK) enzymes. However, the mechanism through which butyrolactone-I is released or produced from the fungal cells during the growth process is still unknown and requires further mechanistic study to unravel this phenomenon [66,67].

The γ-heptalactone that is produced by *A. nidulans* act as QSMs which regulate growth and secondary metabolite production. *A. nidulans* is a well-known filamentous fungus, and produces penicillin as a secondary metabolite. *A. nidulans* produces γ-heptalactone at a higher cell population and has the ability to change the organism’s behavior at a low cell density. This secondary metabolite also facilitates penicillin production. The addition of γ-heptalactone into the culture of wild-type *A. nidulans* strain stimulates the production of penicillin (31.9%) [49].

Raina et al. [46] reported that the addition of γ-butyrolactone molecules (multicolic acid, multicolosic acid, multicolanic acid, and related derivatives) into the *P. sclerotiorums* pent media enhanced the growth of sclerotiorin (6.4-times higher). They concluded that these γ-butyrolactone molecules act as QSMs which trigger the genes involved in the production of sclerotiorin.

### 3.6. Lipids

**Figure 5:**
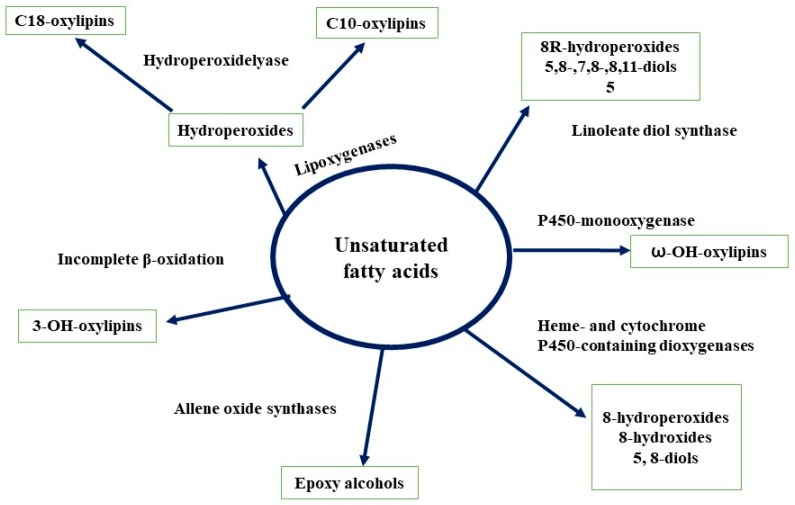
Fungal biosynthesis of oxylipins (adapted from [68]).

Oxylipins are oxygenated fatty acids, that are abundantly present in almost all organisms. Oxylipins in fungi play several functions such as differentiation, growth regulation, reproduction (sexual and asexual), secondary metabolites production (antibodies and mycotoxins), and as QSMs [50,68]. The biosynthesis of oxylipin in fungi is not well characterized compared to that of mammals and plants. However, several new identification techniques and analytical methods make it easy to detect the key enzymes (lipoxygenases, dioxygenases, hydroperoxide lyases, etc.) involved in the synthesis of oxylipins. The detail of the biosynthesis of fungal oxlipins is depicted in Figure 5 [68]. Oxylipins synthesis and secretion in fungi cells represents the putative role of these molecules as QSMs. The *ppo* genes and *psi* factors (oleic, linoleic, and linolenic acids derivatives) are the key genes which facilitate the production of oxylipins in *A. nidulans* [69]. Tsitsigiannis et al. [70] observed that the activation of these genes results in not only the inhibition of the corresponding enzymes but also influences the reproduction type (asexual or sexual) and biosynthesis of mycotoxin. Moreover, they also observed that addition or overexpression of *psiBα* or *psiCα* increased the reproduction of fungal cultures. However, *psiAα* or *psiBβ* caused an increase in asexual reproduction. In another study, Sebolai et al. [71] reported that oxylipin (3-hydroxylated PUFA derivatives) accumulated in the capsules of *C. neoformans* and discharged these compounds via tubular outgrowths into the environment. They predicted that these compound may act as virulence factors of the fungus. Previously, Smith et al. [72] also observed similar oxylipins in *D. uninucleata* during the sexual process. Brown et al. [44] demonstrated that during the development of *A. flavus,* conidia and sclerotia ratio production was influenced by various oxylipins. The addition of oxylipin precursor (linolenic acid) into *A. terreus* culture stimulated the lovastatin biosynthesis process by activating the responsible genes (*lovB* and *lovF*) [45].

Numerous scientists considered the possibility that PUFAs (polyunsaturated fatty acids) may be act as QSMs because PUFAs influence the growth of various fungal species. For example, linoleic acid accelerates the growth of *Monilinia fructicola* [73]. Acceleration of the development of fruiting bodies through unsaturated fatty acids was observed in *Ceratostomella ulmi* and *Nectria haematococca* [74]. The development of perithecia in *N. crassa* was stimulated through linolenic, linoleic, and palmitoleic acids. This effect was only in unsaturated fatty acids whereas not in saturated fatty acids. The transformation of *Ustilago maydis* fungus into filamentary form is caused by PUFAs [75,76]. Moreover, PUFAs hamper cleistothecia or sporulation development in *Aspergillus species* and *Cladosporium caryigenum* [48].

Eicosanoids also belonging to the oxylipin family consist of a 20-carbon backbone. *C. albicans* produced prostaglandin (PG) E2 from exogenous arachidonic acid. This molecule (PGE2) is also produced by humans, and it was observed that fungal PGE2 is in competition with human PG, affecting the host’s immune response [77,78].

The number of studies regarding QSMs in fungal species is limited. Discovering new QSMs in other fungal species is imperative to understand inter and intra kingdom communication. Furthermore, QSMs are produced in very small amounts (nM–μM) and scientists face experimental challenges with regard to the identification, characterization, structural elucidation and purification of these molecules. Scale up of fermentation or molecular biology tools will prove useful way to increase their production [2].

## 4. Fungal Quorum Sensing Inhibitors (QSIs) with Potential Antifungal Activities

**Figure 6:**
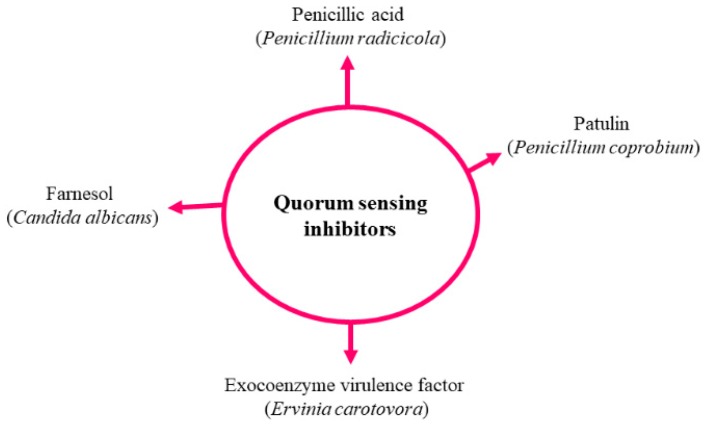
Reported quorum sensing inhibitors.

**Figure 7:**
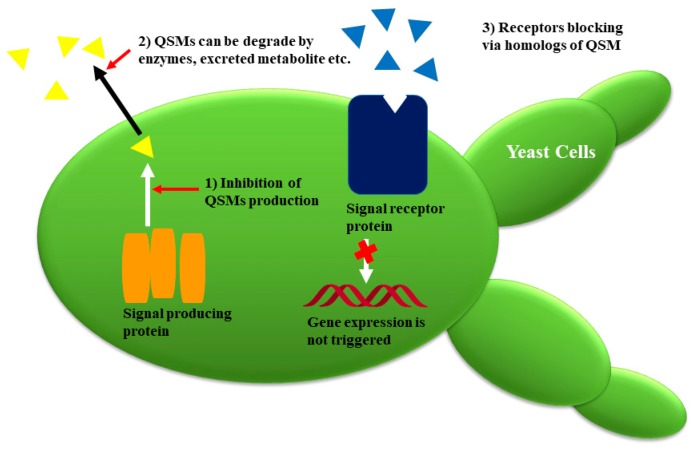
Strategies of fungal quorum-sensing inhibitors for tackling quorum sensing (QS) in pathogenic organisms (adapted from [3]).

In recent years, scientists have attempted to elucidate the molecular mechanism of inhibiting the QS in microorganisms. Regardless of QSMs, the common mechanism involves the synthesis of signals that are released outside the cell via active transport or diffusion. When QSMs reach high concentrations, they bind with the receptor, and subsequently, link with promotor sequences and activate the transcriptional regulators of the specific gene. QS can be hampered in three ways, e.g., (1) by retarding the production of QSMs; (2) these QSMs can be degraded by enzymes; (3) receptors are blocked via homologs of QSMs (Figure 6 and Figure 7) [1,3].

Fungi have the ability to live in many habitats and interact with other organisms (microbes, animals, plants, etc.). Therefore, they have effectively developed a natural mechanism to deal with other organisms by producing secondary metabolites, enzymes and chemicals. Indeed, mycorrhiza and rhizosphere fungi closely interact with bacteria in the soil. Due to this specific trait, they have a natural mechanism to combat population by bacteria for many reasons, e.g., space, nutrition, pathogenicity, etc. They also discharge some metabolites (enzyme, chemicals mycotoxins, etc.) which act as QSIs [3,79,80]. The characteristics of some important QSIs produced by fungi are discussed below.

### 4.1. Farnesol

Farnesol, a secondary metabolite excreted by many dimorphic yeasts (detail describe above section QSMs) also act as a QSI. It has a broad spectrum of anti-microbial potential, e.g., *Fusarium graminearum*, *non-albicans Candida species, Paracoccidioides brasiliensis, C. neoformans*, etc. [81,82,83,84,85]. It also acts as an additive effect toward *S. epidermidis* when used with antibiotics by disturbing its biofilm matrix [86,87]. Various studies reported that a high concentration of farnesol inhibited the formation of the biofilm or new cells inside the biofilm (the detail of the antimicrobial potential described below is given in a separate section) [88].

### 4.2. Others Fungal QSIs

In another study, Zhu et al. [89] demonstrated that *Auricularia auricular* (fruiting body), which contains many heterocyclic compounds (cysteinyldopas, leucodopachrome and dopaquinone), inhibited N-acylhomoserine lactone (AHL) production. Similarly, *Ganoderma lucidum*, *Phellinus igniarius*, *F. graminearum* and *Lasiodiplodia* species also have potential to discharge QSIs [89,90,91]. However, the key compound and mechanism of action have not yet been reported. Rasmussen et al. [92] reported that around 33 *Penicillium* species produced QSIs (penicillic acid and patulin). 

Previously, Petrović et al. [93] reported that methanolic extract of *Agrocybe aegerita* mushroom reduced biofilm formation (84.24%) more than the standard drugs, streptomycin and ampicillin (50.60 and 30.84%, respectively). Another study, conducted by Soković et al. [94] also demonstrated that hot water *Agaricus blazei* extract had strong anti-QS activity at a concentration of 200 µg/mL. Fernandes et al. [95] and Kostić et al. [96] also reported the anti-QS potential of *Polyporus squamosus* and *Armillaria mellea* extract.

### 4.3. The Antifungal Potential of QSMs/QSIs

The mortality rate due to fungal infections significantly increases every year. It is estimated that around more than one million people’s deaths every year occur due to fungal infection. *Candida, Aspergillus* and *Cryptococcus* are common fungal species directly associated with invasive fungal infections [97,98,99,100,101,102,103]. A few classes of antifungal drugs (azoles, polyenes, echinocandins, flucytosine, and allylamines) are available in the market to cope with fungal infections. Woefully, all these drugs possess some side effects or limitations related to safety, toxicity, pharmacokinetics, and their spectrum of activity. Moreover, long-term utilization of these drugs results in increased drug resistance. Thus, the search for alternative antifungal drugs with fewer side effects to combat fungal infections is imperative [101,104]. Recently, Su et al. [101] highlighted some pathways for the control of fungal infections such as ATP sulfurylase, aspartate kinase, homoserine dehydrogenase, ROS (reactive oxygen species) production, threonine synthase, mitochondrial phosphate carrier, transcription factor protein (MET4), biofilm formation, methionine synthase, homoserine kinase, homoserine transacetylase, bromodomain (BD), sulfite transporter, homocysteine synthase, phosphopantetheinyl transferase, and acetolactate synthase. It was also reported that fungal QSMs significantly affect ROS production, biofilm formation, and may be used for the treatment of fungal infections. Mostly, this study focused on farnesol due to its broad spectrum of antimicrobial potential. It showed promising results against various fungus species, e.g., *C. albicans, Aspergillus niger, S. cerevisiae, F. graminearum, P. brasiliensis, A. flavus, A. fumigatus*, and *A. nidulans* [105,106,107,108,109,110,111]. 

High concentrations of farnesol prevent bacterial biofilm formation whereas moderate levels (~25 µM) upregulate microcolony development and biofilm formation in *Streptococcus* mutants. Moreover, in the presence of *S. mutants*, a decrease in farnesol production in *C. albicans* was also observed. Therefore, this interaction of both fungal species by QS may stimulate mixed biofilm formation in oral plaque biofilm. Farnesol also protects against oral Candidiasis and has a synergistic effect with the antifungal drug (fluconazole) [112,113].

Farnesol retards the growth of *S. cerevisiae* by reducing the diacylglycerol (DAG) level, and suppressing the G1 stage of the cell cycle [114,115]. Farnesol was also reported to inhibit *A. nidulans* by ROS (reactive oxygen species) formation [116]. In case of *A. fumigatus*, farnesol disturbed the signaling pathway related to cell wall integrity which further led to the mislocalization of Rho protein that disturbed the hyphal morphology from proliferating [110]. Farnesol was also reported to inhibit *C. parapsilosis* due to overexpression of genes related with aging. Farnesol also negatively affect the genes involved in sterol metabolism, oxidation-reduction, and biofilm formation [117]. It also inhibits the formation of hypha in *C. dubliniensis* [118]. Farnesol also retarded the *P. brasiliensis* growth at higher concentrations whereas it suppresses yeast to hyphae conversion at low concentrations [109].

Farnesol significantly reduced biofilm formation of *C. parapsilosis* when used in combination with echinocandins (caspofungin and micafungin) [119]. Moreover, farnesol also takes part in sterol biosynthesis/stimulating apoptosis by ROS accumulation to damage cellular compartments [120]. Farnesol also displayed antagonistic effects when used in combination with terbinafine/itraconazole, whereas it displayed synergistic effects with fluconazole/5-flurocytosine against *C. albicans*-resistant strains’ biofilm formation [121]. More recently, the same research group observed that this potential antifungal activity toward *C. albicans* was due to regulation of *CYR1* and *PDE2* gene expression which suppresses biofilm formation [122].

Besides farnesol, another QSM (tyrosol) also exhibited antifungal activity alone or in combination with farnesol/standard drugs. For example, Monteiro et al. [55] reported that tyrosol prevented from denture stomatitis caused by *Candida* species. In detail, tyrosol significantly reduced (1.74 to 3.64 log_10_ CFU/cm^2^) the number of adhered cells numbers to the acrylic surface in a mixed and single culture of *C. albicans* and *C. glabrata.* Similarly, in another study, tyrosol was screened against single and mixed biofilm formation of various strains, *C. glabrata ATCC* 90030, and *C. albicans* ATCC 10231 on hydroxyapatite (HA) and acrylic resin surfaces. Their results divulge that tyrosol significantly inhibited biofilm formation against oral pathogenic organisms [123]. Tyrosol showed promising results when used in combination with chlorhexidine gluconate against *C. albicans* by reducing the number of hyphae [124]. Interestingly, it also showed the potent inhibitory activity of *C. albicans* when used with farnesol and may be used for the development of oral care products [125].

Tyrosol also showed potent inhibitory activity against dimorphic fungal species (*Coccidioides posadasii* and *Histoplasma capsulatum*) via leakage with the intracellular molecules [126].

## 5. Concluding Remarks

In recent years, the prevalence of multidrug resistance (MDR) by pathogenic microorganisms against existing antibiotics has increased, which seriously threatens to not only result in immune-compromised patients but also turn into a global public health crisis [127,128]. The eradication of fungal infections is very difficult because of the ubiquitous resistance of pathogens and related shortcomings in the development of conventional drugs, thereby stimulating the scientific community to pursue effective anti-infective strategies with the help of new combinative adjuvant approaches in addition to subsisting antibiotic therapy [129].

Under such circumstances, quorum sensing (QS) will be of great advantage in response to targeting highly complex and evolutionary fungi cell–cell communication phenomena. QS in fungi are well studied in some yeasts (*C. albicans* and *S. cerevisiae*) and fungi (*H. capsulatum, C. ulmi*, and *N. crassa*). The QS on yeast *C. albicans* is the most extensively studied due to its importance as a human pathogen. Although QS in fungi is not uncommon, there is a paucity of information regarding QSMs in fungi except for farnesol and tyrosol. Besides QSMs, fungi also produced secondary metabolites known as QSIs, which act as anti-microbial agents and may be used as broad-spectrum antibiotics for specific diseases. Research in this area is rare and restricted to the laboratory but the results are practically valid. The restriction or control of microbial growth affected by QSS will be beneficial to multiple fields, e.g., agriculture, medicine, and food technology [3].
